# *IRGM* Variants and Susceptibility to Inflammatory Bowel Disease in the German Population

**DOI:** 10.1371/journal.pone.0054338

**Published:** 2013-01-24

**Authors:** Jürgen Glas, Julia Seiderer, Stephanie Bues, Johannes Stallhofer, Christoph Fries, Torsten Olszak, Eleni Tsekeri, Martin Wetzke, Florian Beigel, Christian Steib, Matthias Friedrich, Burkhard Göke, Julia Diegelmann, Darina Czamara, Stephan Brand

**Affiliations:** 1 Department of Medicine II - Grosshadern, Ludwig-Maximilians-University, Munich, Germany; 2 Department of Preventive Dentistry and Periodontology, Ludwig-Maximilians-University, Munich, Germany; 3 Department of Human Genetics, Rheinisch-Westfälische Technische Hochschule, Aachen, Germany; 4 Department of Pediatrics, Hannover Medical School, Hannover, Germany; 5 Max-Planck-Institute of Psychiatry, Munich, Germany; University of Chicago, United States of America

## Abstract

**Background & Aims:**

Genome-wide association studies identified the autophagy gene *IRGM* to be strongly associated with Crohn's disease (CD) but its impact in ulcerative colitis (UC), its phenotypic effects and potential epistatic interactions with other IBD susceptibility genes are less clear which we therefore analyzed in this study.

**Methodology/Principal Findings:**

Genomic DNA from 2060 individuals including 817 CD patients, 283 UC patients, and 961 healthy, unrelated controls (all of Caucasian origin) was analyzed for six *IRGM* single nucleotide polymorphisms (SNPs) (rs13371189, rs10065172 = p.Leu105Leu, rs4958847, rs1000113, rs11747270, rs931058). In all patients, a detailed genotype-phenotype analysis and testing for epistasis with the three major CD susceptibility genes *NOD2*, *IL23R* and *ATG16L1* were performed.

Our analysis revealed an association of the *IRGM* SNPs rs13371189 (p = 0.02, OR 1.31 [95% CI 1.05–1.65]), rs10065172 = p.Leu105Leu (p = 0.016, OR 1.33 [95% CI 1.06–1.66]) and rs1000113 (p = 0.047, OR 1.27 [95% CI 1.01–1.61]) with CD susceptibility. There was linkage disequilibrium between these three *IRGM* SNPs. In UC, several *IRGM* haplotypes were weakly associated with UC susceptibility (p<0.05). Genotype-phenotype analysis revealed no significant associations with a specific IBD phenotype or ileal CD involvement. There was evidence for weak gene-gene-interaction between several SNPs of the autophagy genes *IRGM* and *ATG16L1* (p<0.05), which, however, did not remain significant after Bonferroni correction.

**Conclusions/Significance:**

Our results confirm *IRGM* as susceptibility gene for CD in the German population, supporting a role for the autophagy genes *IRGM* and *ATG16L1* in the pathogenesis of CD.

## Introduction

Crohn's disease (CD) and ulcerative colitis (UC) are chronic inflammatory bowel diseases (IBD) resulting from an inappropriate immune response to microbial antigens in genetically susceptible individuals [1], [2], [3], [4]. Recent genome-wide association studies (GWAS) have provided valuable insights into the genetic architecture particularly of CD, identifying more than 70 CD susceptibility variants with the most significant findings in the gene regions of *NOD2*, *IL23R* and *ATG16L1* [5], [6], [7], [8], [9]. These genetic findings confirm an important role for innate immunity, proinflammatory IL-23/Th17 immune responses as well as autophagy for both gut homeostasis and the development of chronic inflammation in IBD.

In addition to the CD susceptibility gene *ATG16L1* involved in autophagy [9], [10], [11], recent GWAS identified the single nucleotide polymorphism (SNP) rs13361189 – a SNP lying immediately upstream of the autophagy gene *IRGM* (immunity-related GTPase family M) – and other *IRGM* SNPs to be strongly associated with CD [12], [13]. Since the discovery of the *IRGM* as a CD susceptibility gene, further studies have investigated *IRGM* gene variants in both adult and pediatric CD [14], [15], [16], [17], [18], [19], [20], [21], [22] as well as in UC [14], confirming its role in the IBD pathogenesis.

A functional study suggested a common, 20-kb deletion polymorphism upstream of *IRGM*, which is in perfect linkage disequilibrium (LD) with rs13361189, as a likely causal variant, since the deletion allele modulated the expression of IRGM in transformed cells [22]. Another recent study implicated a variant in the 5′-untranslated region (−308(GTTT)_5_) to be independently associated with CD [20], while the very recent study by Brest et al. [21] demonstrated functional effects of the synonymous SNP rs10065172 (c.313C>T), which is also in linkage disequilibrium with rs13361189 and the deletion polymorphism. This exonic, synonymous variant rs10065172 in *IRGM* alters a binding site for certain microRNAs (miR-196) and causes deregulation of IRGM-dependent xenophagy of bacteria in patients with CD [21], therefore suggesting rs10065172 as disease-causing variant.

These studies implicate that autophagy plays an important role in human inflammatory disorders by direct elimination of intracellular bacteria and activation of pattern recognition receptor (PRR) signaling which is involved in gut homeostasis and CD pathogenesis [10], [23]. The *IRGM* gene belongs to immunity-related GTPases (IRG), a family of genes in mammalian species induced by interferons (IFNs) and functioning as key mediators of IFN-regulated resistance to intracellular bacteria and protozoa [23]. IRGM has been shown to play a role in the autophagy-targeted destruction of *Mycobacterium bovis* BCG [23] and the IFN-γ-induced host defense against *Salmonella typhimurium* infection [10]. Interestingly, a recent study in CD patients demonstrated that autophagy limits the replication of intracellular adherent-invasive *Escherichia coli* (AIEC) associated with ileal CD and that *IRGM*- and *ATG16L1*-deficient cells had enhanced intracellular AIEC bacteria replication, suggesting a significant impact on the outcome of intestinal inflammation [24].

While several GWAS and replication studies established *IRGM* as a CD susceptibility gene, its effects on the IBD phenotype are less clear. In addition, epistatic interactions with other IBD susceptibility genes, in particular the second autophagy gene *ATG16L1*, have not been studied in detail. Therefore, in this study, we aimed to analyze the role of *IRGM* on CD and UC susceptibility as well as its effect on the IBD phenotype in a large patient-control cohort. In addition, we performed a detailed epistasis analysis of *IRGM* with the three major CD susceptibility genes *NOD2*, *ATG16L1* and *IL23R*. In total, six major *IRGM* SNPs, for which associations with CD were shown in previous studies (see details in Methods), were genotyped in more than 2000 German IBD patients and controls.

## Patients and Methods

### Ethics statement

The study was approved by the Ethics committee of the Medical Faculty of the Ludwig-Maximilians-University Munich. Written, informed consent was obtained from all patients prior to the study. Study protocols were based on the ethical principles for medical research involving human subjects of the Helsinki Declaration (http://www.wma.net/e/policy/b3.htm).

### Study population and definition of IBD phenotype

The study population (n = 2060) consisted of 1099 IBD patients including 817 patients with CD, 283 patients with UC, and 961 healthy, unrelated controls, all of Caucasian origin. Patient charts were analyzed for demographic and clinical parameters (disease behaviour and anatomic location of IBD, disease-related complications, history of surgery or immunosuppressive therapy) and all patients participated in a detailed questionnaire including an interview at time of enrolment. The diagnosis of CD or UC was determined according to endoscopic, histopathologic and radiological criteria of current international guidelines [25]. Patients with clinical features of both CD and UC (and therefore classified as “indeterminate colitis”) were excluded from this study. Patients with CD were assessed based on the Montreal classification including age at diagnosis (A), location (L), and behaviour (B) of disease [26]. In patients with UC, anatomic location was also assessed following the Montreal classification analyzing the criteria ulcerative proctitis (E1), left-sided UC (distal UC; E2), and extensive UC (pancolitis; E3) [26]. The demographic baseline characteristics of the study population were collected blind to the results of the genotype analyses and are summarized in Table 1.

**Table 1:** Demographic characteristics of the IBD study population.

	Crohn's disease	Ulcerative colitis	Controls
	*n = 817*	*n = 283*	*n = 961*
**Gender**			
Male (%)	46.0	53.0	63.6
Female (%)	54.0	47.0	36.4
**Age** (yrs)			
Mean ± SD	40.7±13.3	43.8±14.4	47.3±9.06
Range	15–81	16–88	19–68
**Body mass index**			
Mean ± SD	23.0±4.2	23.9±4.5	
Range	13–41	15–54	
**Age at diagnosis** (yrs)			
Mean ± SD	27.9±12.0	31.3±13.7	
Range	6–78	4–81	
**Disease duration** (yrs)			
Mean ± SD	13.1±8.8	11.9±8.4	
Range	0–46	1–50	
**Positive family history of IBD** (%)	16.7	17.4	

### DNA extraction

From all study participants, blood samples were taken and genomic DNA was isolated from peripheral blood leukocytes using the DNA blood mini kit from Qiagen (Hilden, Germany) according to the manufacturer's guidelines.

### Genotyping of the IRGM variants

Six *IRGM* SNPs (rs13361189, rs10065172 = pLeu105Leu, rs4958847, rs1000113, rs931058, rs11747270) were genotyped. The selection of these six *IRGM* SNPs was based on previous studies showing associations for these SNPs in large case-control cohorts. The SNPs rs13361189, rs10065172 = p.Leu105Leu and rs4958847 were selected from the study of Parkes et al. [12], while the SNPs rs1000113 and rs931058 were tested in the study of the Wellcome Trust Case Control Consortium (WTCCC) [13]. The SNPs rs13361189 and rs10065172 = p.Leu105Leu served also as proxies for a common, 20-kb deletion polymorphism upstream of *IRGM*, since they are in perfect linkage disequilibrium (r^2^ = 1.0) with this deletion polymorphism [22]. Additionally rs11747270, which was the most strongly CD-associated SNP within the *IRGM* region in the meta-analysis of Barrett et al., was included.

*IRGM* genotyping was performed by PCR and melting curve analysis using a pair of fluorescence resonance energy transfer (FRET) probes in a LightCycler® 480 Instrument (Roche Diagnostics, Mannheim, Germany) as previously described in detail [27], [28], [29], [30], [31]. The donor fluorescent molecule (fluorescein) at the 3′-end of the sensor probe is excited at its specific fluorescence excitation wavelength (533 nm) and the energy is transferred to the acceptor fluorescent molecule at the 5′-end (LightCycler Red 610, 640 or 670) of the anchor probe. The specific fluorescence signal emitted by the acceptor molecule is detected by the optical unit of the LightCycler 480 instrument. The sensor probe is exactly matching to one allele of each SNP, preferentially to the rarer allele, whereas in the case of the other allele there is a mismatch resulting in a lower melting temperature. The total volume of the PCR was 5 µl containing 25 ng of genomic DNA, 1× Light Cycler 480 Genotyping Master (Roche Diagnostics), 2.5 pmol of each primer and 0.75 pmol of each FRET probe (TIB MOLBIOL, Berlin, Germany). In the case of rs11747270, the amount of the forward primer was reduced to one fifth and in the case of rs4958847 the reverse primer was reduced to one half. In the case of rs10065172 and rs931058, the reverse primers were reduced to one third, respectively. The PCR comprised an initial denaturation step (95°C for 10 min) and 45 cycles (50 cycles in the case of rs10065172) [95°C for 10°C sec, 60°C (55°C in the case of rs10065172) for 10 sec, 72°C for 15 sec]. The melting curve analysis comprised an initial denaturation step (95°C for 1 min), a step rapidly lowering the temperature to 40°C and holding for 2 min, and a heating step slowly (1 acquisition/°C) increasing the temperature up to 95°C and continuously measuring the fluorescence intensity. The results of melting curve analysis have been confirmed by analyzing two patient samples for each possible genotype using sequence analysis. For sequencing, the total volume of the PCR was 100 µl containing 250 ng of genomic DNA, 1× PCR buffer (Qiagen, Hilden, Germany), a final MgCl_2_ concentration of 2 mM, 0.5 mM of a dNTP mix (Sigma, Steinheim, Germany), 2.5 units of HotStar Plus Taq™ DNA polymerase (Qiagen) and 10 pmol of each primer (TIB MOLBIOL). The PCR comprised an initial denaturation step (95°C for 5 min), 35 cycles (denaturation at 94°C for 30 sec, primer annealing at 60°C for 30 sec, extension at 72°C for 30 sec) and a final extension step (72°C for 10 min). The PCR products were purified using the QIAquick PCR Purification Kit (Qiagen) and sequenced by a commercial sequencing company (Sequiserve, Vaterstetten, Germany). All sequences of primers and FRET probes used for genotyping and for sequence analysis are given in Tables S1 and S2.

### Genotyping of NOD2, IL23R and ATG16L1 variants

For analysis of potential epistatic interactions, the genotypes of gene variants in *NOD2*, *IL23R* and *ATG16L1* were available from previous studies for all patients and controls analyzed [11], [27], [32], [33], [34], [35], [36]. Genotyping of the *NOD2* variants p.Arg702Trp (rs2066844), p.Gly908Arg (rs2066847), and p.Leu1007fsX1008 (rs2066847) was performed as described previously (primer sequences available on request) [34]. The 10 *IL23R* SNPs (rs1004819, rs7517847, rs10489629, rs2201841, rs11465804, rs11209026 = p.Arg381Gln, rs1343151, rs10889677, rs11209032, rs1495965) and nine *ATG16L1* SNPs (rs13412102, rs12471449, rs6431660, rs1441090, rs2289472, rs2241880 ( = T300A), rs2241879, rs3792106, rs4663396) were genotyped by PCR and melting curve analysis as described previously [11], [35].

### Statistical analyses

For evaluation of data, the SPSS 13.0 software (SPSS Inc., Chicago, IL, USA) and R-2.13.1 (http://cran.r-project.org) were used. Each genetic marker was tested for Hardy-Weinberg equilibrium in the control group. Fisher's exact test was used for comparison between categorical variables and Student's t test was applied for quantitative variables. All tests were two-tailed and p-values<0.05 were considered as significant. Odds ratios were calculated for the minor allele of each SNP. Correction for multiple testing was performed by Bonferroni correction where indicated. Haplotype analysis was calculated using the –hap-logistic command in PLINK (http://pngu.mgh.harvard.edu/~purcell/plink/), epistasis analysis was performed with the –epistasis option. LD between SNPs was evaluated using the R-library genetics. Genotype-phenotype associations were also tested in R using logistic regression.

## Results

### The IRGM gene variants are associated with susceptibility to CD

The allele frequencies of the SNPs rs13371189, rs10065172 = p.Leu105Leu, rs4958847, rs11747270, rs931058 and rs1000113 of all three subgroups (CD, UC, and controls) were in accordance with the predicted Hardy-Weinberg equilibrium and are summarized in Table 2. Overall, our analysis revealed an association of the *IRGM* variants rs13371189 (p = 0.02, OR 1.31 [95% CI 1.05–1.65]), rs10065172 = p.Leu105Leu (p = 0.016, OR 1.33 [95% CI 1.06–1.66]) and rs1000113 (p = 0.047, OR 1.27 [95% CI 1.01–1.61]) with the susceptibility to CD. Similar to previous studies, rs13371189 and rs10065172 = p.Leu105Leu were in perfect linkage disequilibrium (r^2^≈1.0) in all three subgroups (CD, UC, controls; Tables S3, S4, S5). Strong linkage disequilibrium was also shown for these two SNPs with the third CD-associated *IRGM* SNP rs1000113 (Tables S3, S4, S5). With exception of rs11747270, none of the genotyped *IRGM* SNPs was associated with UC susceptibility (Table 2).

**Table 2:** Associations of *IRGM* gene markers in CD and UC case-control association studies.

*IRGM* SNP	Genotype/Allele		Crohn's disease			Ulcerative colitis		Controls
			*n = 815*			*n = 283*		*n = 961*
		Genotype/allele frequency	p-value	OR [95% CI]	Genotype/allele frequency	p-value	OR [95% CI]	Genotype/allele frequency
**rs13361189**	TT	0.799	**0.048**		0.833	0.060		0.840
	TC	0.191		**1.29 [1.00–1.67]**	0.149		0.97 [0.65–1.41]	0.156
	CC	0.010		2.47 [0.66–11.27]	0.018		4.28 [0.91–21.7]	0.004
	T	0.895			0.908			0.918
	C	0.105	**0.020**	**1.31 [1.05–1.65]**	0.092	0.440	1.13 [0.82–1.57]	0.082
**rs10065172**	CC	0.799	**0.050**		0.834	0.125		0.842
** = p.Leu105Leu**	CT	0.191		**1.32 [1.02–1.70]**	0.148		0.98 [0.65–1.43]	0.153
	TT	0.010		2.44 [0.65–11.14]	0.018		4.20 [0.90–21.36]	0.005
	C	0.894			0.908			0.918
	T	0.106	**0.017**	**1.33 [1.06–1.66]**	0.092	0.438	1.14 [0.82–1.58]	0.082
**rs4958847**	GG	0.745	0.205		0.791	0.774		0.778
	GA	0.235		1.23 [0.97–.155]	0.184		0.90 [0.63–1.28]	0.201
	AA	0.020		0.98 [0.47–2.01]	0.025		1.16 [0.41–2.91]	0.021
	G	0.863			0.883			0.879
	A	0.137	0.159	1.16 [0.95–1.41]	0.117	0.825	0.96 [0.72–1.28]	0.121
**rs1000113**	CC	0.813	0.119		0.848	0.174		0.849
	CT	0.179		1.27 [0.98–1.65]	0.138		0.94 [0.62–1.39]	0.147
	TT	0.008		1.84 [0.42.8.90]	0.014		3.38 [0.62–18.28]	0.004
	C	0.903			0.917			0.922
	T	0.097	**0.048**	**1.27 [1.01–1.61]**	0.083	0.658	1.08 [0.76–1.51]	0.078
**rs11747270**	AA	0.885	0.643		0.873	**0.033**		0.899
	AG	0.109		1.14 [0.80–1.60]	0.108		1.14 [0.69–1.82]	0.097
	GG	0.006		1.55 [0.26–10.66]	0.019		**5.34 [1.03–34.64]**	0.004
	A	0.940			0.927			0.948
	G	0.060	0.385	1.16 [0.85–1.57]	0.073	0.084	1.42 [0.96–2.12]	0.052
**rs931058**	AA	0.829	0.632		0.865	0.084		0.845
	AT	0.162		1.10 [0.84–1.44]	0.117		0.76 [0.49–1.15]	0.149
	TT	0.009		1.40 [0.40–5.05]	0.018		2.74 [0.66–10.89]	0.006
	A	0.910			0.924			0.919
	T	0.090	0.365	1.12 [0.88–1.42]	0.076	0.792	0.94 [0.66–1.33]	0.081

Note: Genotype and allele frequencies, p-values, and odds ratios (OR, shown for the minor allele) with 95% confidence intervals (CI) are depicted for both the CD and UC case-control cohorts. rs13361189 and rs10065172 ( = p.Leu105Leu) are in linkage disequilibrium. The minor differences in p-values and ORs are related to small differences regarding the genotyping success rates of both SNPs resulting in small differences of patients included.

### IRGM haplotype analysis

Next, we performed a detailed haplotype analysis investigating the role of *IRGM* haplotypes on CD and UC susceptibility. As demonstrated in tables 3 and 4, several *IRGM* haplotypes demonstrated an association with CD and UC susceptibility. In CD, the strongest associations were found for haplotypes containing at least one of the most strongly CD-associated SNP rs13361189 or rs10065172 (Table 3), while in UC, the strongest association was found for rs11747270-rs931058 (omnibus p-value 1.57×10^−2^) (Table 4). However, given the large number of haplotypes analyzed, none of these associations withstood Bonferroni correction for multiple testing.

**Table 3:** Haplotypes of *IRGM* SNPs in CD case-control sample and omnibus p-values for association with CD susceptibility.

Haplotype combination	Haplotype	Haplotype frequency	P-value	OR (95% CI)
rs13361189-rs4958847	CA	0.09	**2.36×10^−2^**	1.31 [1.04–1.65]
	TA	0.04	3.60×10^−1^	0.85 [0.60–1.20]
	TG	0.87	1.36×10^−1^	0.86 [0.71–1.05]
rs13361189-rs1000113	CT	0.09	**2.96×10^−2^**	1.30 [1.03–1.65]
	TC	0.91	**1.92×10^−2^**	0.76 [0.60–0.96]
rs13361189-rs11747270	CG	0.06	2.76×10^−1^	1.19 [0.87–1.63]
	CA	0.02	8.33×10^−1^	0.95 [0.60–1.50]
	TA	0.92	5.31×10^−1^	0.92 [0.71–1.20]
rs13361189-rs931058	CT	0.07	1.55×10^−1^	1.21 [0.93–1.57]
	TT	0.01	4.49×10^−1^	0.80 [0.45–1.42]
	CA	0.02	**2.14×10^−2^**	1.72 [1.08–2.73]
	TA	0.90	**4.90×10^−2^**	0.81 [0.65–1.00]
rs4958847-rs1000113	AT	0.09	3.93×10^−1^	1.28 [1.01–1.62]
	AC	0.04	6.27×10^−1^	0.92 [0.67–1.27]
	GC	0.87	1.67×10^−1^	0.87 [0.72–1.06]
rs4958847-rs11747270	AG	0.06	3.13×10^−1^	1.17 [0.86–1.59]
	AA	0.06	5.16×10^−1^	0.91 [0.68–1.21]
	GA	0.88	8.94×10^−1^	0.99 [0.79–1.23]
rs4958847-rs931058	AT	0.07	1.95×10^−1^	1.19 [0.91–1.55]
	GT	0.01	5.22×10^−1^	0.82 [0.45–1.49]
	AA	0.06	4.44×10^−1^	1.12 [0.84–1.50]
	GA	0.86	2.23×10^−1^	0.89 [0.74–1.07]
rs1000113-rs11747270	TG	0.05	2.87×10^−1^	1.19 [0.86–1.64]
	TA	0.02	4.08×10^−1^	0.82 [0.51–1.31]
	CA	0.92	7.81×10^−1^	0.96 [0.74–1.26]
rs1000113-rs931058	TT	0.07	1.35×10^−1^	1.22 [0.94–1.58]
	CT	0.01	2.44×10^−1^	0.71 [0.40–1.26]
	TA	0.02	9.43×10^−1^	1.60 [0.92–2.77]
	CA	0.90	1.39×10^−1^	0.85 [0.68–1.06]
rs11747270-rs931058	GT	0.04	4.96×10^−1^	1.13 [0.79–1.61]
	AT	0.03	7.98×10^−2^	0.70 [0.47–1.04]
	GA	0.01	5.42×10^−1^	1.21 [0.66–2.23]
	AA	0.91	6.87×10^−1^	1.05 [0.83–1.33]
rs13361189-rs4958847-rs1000113	CAT	0.08	**2.83×10^−2^**	1.31 [1.03–1.67]
	TAC	0.04	3.94×10^−1^	0.86 [0.61–1.22]
	TGC	0.87	1.30×10^−1^	0.86 [0.71–1.05]
rs13361189-rs4958847-rs11747270	CAG	0.06	**1.97×10^−2^**	1.40 [1.06–1.86]
	CAA	0.03	4.42×10^−1^	1.19 [0.76–1.85]
	TAA	0.04	3.61×10^−1^	0.85 [0.60–1.20]
	TGA	0.87	1.58×10^−1^	0.87 [0.72–1.06]
rs13361189-rs4958847-rs931058	CAT	0.07	1.47×10^−1^	1.21 [0.94–1.57]
	TGT	0.01	4.73×10^−1^	0.81 [0.45–1.45]
	CAA	0.02	**3.48×10^−2^**	1.66 [1.04–2.66]
	TAA	0.04	3.78×10^−1^	0.86 [0.61–1.21]
	TGA	0.86	2.10×10^−1^	0.89 [0.74–1.07]
rs13361189-rs1000113-rs11747270	CTG	0.06	**1.47×10^−2^**	1.44 [1.07–1.93]
	CTA	0.03	7.54×10^−1^	1.07 [0.70–1.64]
	TCA	0.91	**2.93×10^−2^**	0.78 [0.62–0.97]
rs13361189-rs1000113-rs931058	CTT	0.07	1.49×10^−1^	1.21 [0.93–1.57]
	TCT	0.01	2.95×10^−1^	0.73 [0.41–1.31]
	CTA	0.01	**4.89×10^−2^**	1.76 [1.00–3.09]
	TCA	0.89	7.37×10^−1^	0.82 [0.66–1.02]
	CGT	0.05	5.34×10^−2^	1.37 [1.00–1.89]
	CAT	0.02	7.99×10^−1^	0.94 [0.57–1.53]
	TAT	0.01	3.43×10^−1^	0.75 [0.42–1.35]
	CGA	0.02	9.93×10^−2^	1.62 [0.91–2.87]
	TAA	0.89	7.46×10^−2^	0.82 [0.67–1.02]
rs4958847-rs1000113-rs11747270	ATG	0.06	**1.66×10^−2^**	1.43 [1.07–1.92]
	ATA	0.03	8.65×10^−1^	1.04 [0.66–1.63]
	ACA	0.04	6.70×10^−1^	0.93 [0.67–1.30]
	GCA	0.87	1.88×10^−1^	0.88 [0.72–1.07]
rs4958847-rs1000113-rs931058	ATT	0.07	1.34×10^−1^	1.22 [0.94–1.58]
	GCT	0.01	3.35×10^−1^	0.75 [0.41–1.35]
	ATA	0.02	9.36×10^−2^	1.60 [0.92–2.77]
	ACA	0.04	7.23×10^−1^	0.94 [0.69–1.30]
	GCA	0.86	2.94×10^−1^	0.91 [0.75–1.09]
	AGT	0.05	**4.87×10^−2^**	1.38 [1.00–1.90]
	AAT	0.02	5.87×10^−1^	0.87[0.53–1.44]
	GAT	0.01	4.64×10^−1^	0.80 [0.44–1.45]
	AGA	0.01	3.39×10^−1^	1.36 [0.72–2.55]
	AAA	0.04	7.02×10^−1^	1.07 [0.76–1.51]
	GAA	0.86	2.59×10^−1^	0.90 [0.75–1.08]
rs1000113-rs11747270-rs931058	TGT	0.05	**4.38×10^−2^**	1.39 [1.01–1.91]
	TAT	0.02	7.83×10^−1^	0.93 [0.57–1.53]
	CAT	0.01	2.51×10^−1^	0.71 [0.40–1.27]
	TGA	0.01	1.51×10^−1^	1.66 [0.83–3.32]
	CAA	0.90	1.77×10^−1^	0.86 [0.69–1.07]
rs13361189-rs4958847-rs1000113-rs11747270	CATG	0.06	**1.83×10^−2^**	1.42 [1.06–1.90]
	CATA	0.03	7.24×10^−1^	1.08 [0.70–1.65]
	TACA	0.04	3.95×10^−1^	0.86 [0.61–1.22]
	TGCA	0.87	1.69×10^−1^	0.87 [0.72–1.06]
rs13361189-rs4958847-rs1000113-rs931058	CATT	0.07	1.45×10^−1^	1.21 [0.94–1.56]
	TGCT	0.01	3.49×10^−1^	0.76 [0.42–1.36]
	CATA	0.01	5.14×10^−2^	1.75 [1.00–3.07]
	TACA	0.04	3.93×10^−1^	0.86 [0.61–1.22]
	TGCA	0.86	2.61×10^−1^	0.90 [0.75–1.08]
	CAGT	0.05	6.44×10^−2^	1.35 [0.98–1.86]
	CAAT	0.02	7.99×10^−1^	0.94 [0.57–1.54]
	TGAT	0.01	3.65×10^−1^	0.76 [0.42–1.37]
	CAGA	0.01	1.35×10^−1^	1.58 [0.87–2.88]
	TAAA	0.03	4.22×10^−1^	0.87 [0.61–1.23]
	TGAA	0.86	2.62×10^−1^	0.90 [0.75–1.08]
	CTGT	0.05	5.06×10^−2^	1.38 [1.00–1.91]
	CTAT	0.02	8.57×10^−1^	0.96 [0.58–1.57]
	TCAT	0.01	2.87×10^−1^	0.73 [0.41–1.31]
	CTGA	0.01	1.09×10^−1^	1.78 [0.88–3.61]
	TCAA	0.89	9.46×10^−2^	0.83 [0.67–1.03]
rs4958847-rs1000113-rs11747270-rs931058	ATGT	0.05	**4.74×10^−2^**	1.39 [1.00–1.92]
	ATAT	0.02	8.13×10^−1^	0.94 [0.57–1.55]
	GCAT	0.01	2.85×10^−1^	0.73 [0.40–1.31]
	ATGA	0.01	1.58×10^−1^	1.65 [0.82–3.31]
	ACAA	0.04	7.92×10^−1^	0.96 [0.68–1.33]
	GCAA	0.86	3.15×10^−1^	0.91 [0.76–1.09]
rs13361189-rs4958847-rs1000113-rs11747270-rs931058	CATGT	0.05	**4.96×10^−2^**	1.38 [1.00–1.90]
	CATAT	0.02	8.65×10^−1^	0.96 [0.58–1.57]
	TGCAT	0.01	2.97×10^−1^	0.73 [0.41–1.31]
	CATGA	0.01	1.07×10^−1^	1.79 [0.88–3.64]
	TACAA	0.04	4.04×10^−1^	0.86 [0.61–1.22]
	TGCAA	0.86	3.07×10^−1^	0.91 [0.76–1.09]

**Note:** P-values<0.05 are depicted in bold (uncorrected p-values). No association remained significant after Bonferroni correction for multiple testing. rs10065172, which is in linkage disequilibrium with rs13361189, was excluded from the haplotype analysis.

**Table 4:** Haplotypes of *IRGM* SNPs in UC case-control sample and omnibus p-values for association with UC susceptibility.

Haplotype combination	Haplo type	Haplo type frequency	P-value	OR (95% CI)
rs13361189-rs4958847	CA	0.09	4.14×10^−1^	1.15 [0.82–1.61]
	TA	0.04	9.62×10^−2^	0.62 [0.35–1.09]
	TG	0.87	7.46×10^−1^	1.05 [0.78–1.41]
rs13361189-rs1000113	CT	0.09	5.73×10^−1^	1.10 [0.79–1.53]
	TC	0.91	5.13×10^−1^	0.90 [0.65–1.24]
rs13361189-rs11747270	CG	0.06	5.83×10^−2^	1.45 [0.99–2.13]
	CA	0.02	8.69×10^−2^	0.49 [0.22–1.11]
	TA	0.92	5.96×10^−1^	0.91 [0.64–1.29]
rs13361189-rs931058	CT	0.07	5.37×10^−1^	1.12 [0.78–1.60]
	TT	0.01	**3.25×10^−2^**	0.11 [0.02–0.83]
	CA	0.02	6.69×10^−1^	1.16 [0.59–2.29]
	TA	0.90	8.13×10^−1^	1.04 [0.75–1.44]
rs4958847-rs1000113	AT	0.09	6.51×10^−1^	1.08 [0.77–1.51]
	AC	0.04	2.69×10^−1^	0.76 [0.46–1.24]
	GC	0.87	7.45×10^−1^	1.05 [0.78–1.41]
rs4958847-rs11747270	AG	0.06	6.10×10^−2^	1.44 [0.98–2.11]
	AA	0.06	**3.21×10^−2^**	0.59 [0.36–0.96]
	GA	0.88	7.25×10^−1^	1.06 [0.77–1.47]
rs4958847-rs931058	AT	0.07	6.10×10^−1^	1.10 [0.76–1.59]
	GT	0.01	5.34×10^−2^	0.24 [0.06–1.02]
	AA	0.06	3.50×10^−1^	0.81 [0.52–1.26]
	GA	0.86	3.79×10^−1^	1.13 [0.86–1.48]
rs1000113-rs11747270	TG	0.05	1.19×10^−1^	1.38 [0.92–2.07]
	TA	0.02	**4.25×10^−2^**	0.41 [0.17–0.97]
	CA	0.92	7.46×10^−1^	0.94 [0.66–1.34]
rs1000113-rs931058	TT	0.07	6.54×10^−1^	1.09 [0.75–1.59]
	CT	0.01	5.92×10^−2^	0.32 [0.10–1.05]
	TA	0.02	9.62×10^−1^	1.02 [0.45–2.30]
	CA	0.90	7.00×10^−1^	1.07 [0.76–1.51]
rs11747270-rs931058	GT	0.04	6.56×10^−2^	1.49 [0.97–2.28]
	AT	0.03	**6.38×10^−3^**	0.34 [0.16–0.74]
	GA	0.01	9.28×10^−1^	1.04 [1.45–2.42]
	AA	0.91	6.76×10^−1^	1.08 [0.75–1.55]
rs13361189-rs4958847-rs1000113	CAT	0.08	5.75×10^−1^	1.10 [0.79–1.53]
	TAC	0.04	1.15×10^−1^	0.63 [0.36–1.12]
	TGC	0.87	7.18×10^−1^	1.05 [0.81–1.37]
rs13361189-rs4958847-rs11747270	CAG	0.06	**4.77×10^−2^**	1.45 [1.00–2.09]
	CAA	0.03	1.02×10^−1^	0.52 [0.24–1.14]
	TAA	0.04	1.06×10^−1^	0.63 [0.35–1.10]
	TGA	0.87	6.83×10^−1^	1.06 [0.80–1.40]
rs13361189-rs4958847-rs931058	CAT	0.07	5.34×10^−1^	1.12 [0.78–1.60]
	TGT	0.01	**4.54×10^−2^**	0.22 [0.05–0.97]
	CAA	0.02	6.01×10^−1^	1.21 [0.59–2.47]
	TAA	0.04	1.02×10^−1^	0.62 [0.35–1.10]
	TGA	0.86	3.22×10^−1^	1.15 [0.87–1.52]
rs13361189-rs1000113-rs11747270	CTG	0.06	7.42×10^−2^	1.42 [0.97–2.09]
	CTA	0.03	6.98×10^−2^	0.47 [0.20–1.06]
	TCA	0.91	6.08×10^−1^	0.92 [0.66–1.27]
rs13361189-rs1000113-rs931058	CTT	0.07	6.34×10^−1^	1.09 [0.76–1.55]
	TCT	0.01	3.66×10^−2^	0.21 [0.05–0.91]
	CTA	0.01	7.89×10^−1^	1.13 [0.46–2.77]
	TCA	0.89	7.94×10^−1^	1.04 [0.77–1.40]
	CGT	0.05	6.01×10^−2^	1.48 [0.98–2.23]
	CAT	0.02	9.74×10^−2^	0.48 [0.20–1.15]
	TAT	0.01	**3.18×10^−2^**	0.14 [0.02–0.84]
	CGA	0.02	6.03×10^−1^	1.25 [0.54–2.90]
	TAA	0.89	7.85×10^−1^	1.04 [0.78–1.38]
rs4958847-rs1000113-rs11747270	ATG	0.06	8.39×10^−2^	1.40 [0.96–2.05]
	ATA	0.03	6.18×10^−2^	0.46 [0.20–1.04]
	ACA	0.04	1.22×10^−1^	0.65 [0.37–1.12]
	GCA	0.87	6.59×10^−1^	1.07 [0.79–1.44]
rs4958847-rs1000113-rs931058	ATT	0.07	6.42×10^−1^	1.09 [0.76–1.57]
	GCT	0.01	**3.92×10^−2^**	0.21 [0.05–0.93]
	ATA	0.02	9.58×10^−1^	1.02 [0.49–2.14]
	ACA	0.04	2.31×10^−1^	0.73 [0.44–1.22]
	GCA	0.86	3.15×10^−1^	1.15 [0.88–1.51]
	AGT	0.05	6.18×10^−2^	1.48 [0.98–2.23]
	AAT	0.02	5.21×10^−2^	0.40 [0.16–1.01]
	GAT	0.01	**4.83×10^−2^**	0.22 [0.05–0.99]
	AGA	0.01	6.52×10^−1^	1.23 [0.50–3.02]
	AAA	0.04	1.87×10^−1^	0.70 [0.41–1.19]
	GAA	0.86	3.10×10^−1^	1.15 [0.88–1.51]
rs1000113-rs11747270-rs931058	TGT	0.05	8.45×10^−2^	1.44 [0.95–2.18]
	TAT	0.02	6.59×10^−2^	0.42 [0.17–1.06]
	CAT	0.01	**3.44×10^−2^**	0.21 [0.05–0.89]
	TGA	0.01	7.36×10^−1^	1.19 [0.43–3.27]
	CAA	0.90	7.21×10^−1^	1.06 [0.77–1.46]
rs13361189-rs4958847-rs1000113-rs11747270	CATG	0.06	7.90×10^−2^	1.41 [0.96–2.07]
	CATA	0.03	7.66×10^−2^	0.47 [0.21–1.08]
	TACA	0.04	1.14×10^−1^	0.63 [0.36–1.12]
	TGCA	0.87	6.60×10^−1^	1.07 [0.79–1.45]
rs13361189-rs4958847-rs1000113-rs931058	CATT	0.07	6.27×10^−1^	1.10 [0.75–1.62]
	TGCT	0.01	**4.16×10^−2^**	0.22 [0.05–0.94]
	CATA	0.01	8.01×10^−1^	1.12 [0.46–2.70]
	TACA	0.04	1.15×10^−1^	0.63 [0.36–1.12]
	TGCA	0.86	2.93×10^−1^	1.16 [0.88–1.53]
	CAGT	0.05	6.41×10^−2^	1.47 [0.98–2.21]
	CAAT	0.02	7.22×10^−2^	0.43 [0.17–1.08]
	TGAT	0.01	**4.31×10^−2^**	0.23 [0.05–0.96]
	CAGA	0.01	4.84×10^−1^	1.35 [0.58–3.13]
	TAAA	0.03	1.18×10^−1^	0.63 [0.36–1.12]
	TGAA	0.86	2.98×10^−1^	1.16 [0.88–1.53]
	CTGT	0.05	8.24×10^−2^	1.45 [0.95–2.20]
	CTAT	0.02	7.52×10^−2^	0.44 [0.17–1.09]
	TCAT	0.01	**3.69×10^−2^**	0.21 [0.05–0.91]
	CTGA	0.01	6.42×10^−1^	1.28 [0.45–3.63]
	TCAA	0.89	7.23×10^−1^	1.06 [0.77–1.46]
rs4958847-rs1000113-rs11747270-rs931058	ATGT	0.05	8.41×10^−2^	1.44 [0.95–2.18]
	ATAT	0.02	7.51×10^−2^	0.44 [0.17–1.09]
	GCAT	0.01	**3.55×10^−2^**	0.21 [0.05–0.90]
	ATGA	0.01	7.47×10^−1^	1.19 [0.41–3.43]
	ACAA	0.04	1.43×10^−1^	0.66 [0.38–1.15]
	GCAA	0.86	2.76×10^−1^	1.17 [0.88–1.55]
rs13361189-rs4958847-rs1000113-rs11747270-rs931058	CATGT	0.05	7.90×10^−2^	1.45 [0.96–2.19]
	CATAT	0.02	7.67×10^−2^	0.44 [0.17–1.09]
	TGCAT	0.01	**3.76×10^−2^**	0.21 [0.05–0.92]
	CATGA	0.01	6.37×10^−1^	1.28 [0.46–3.57]
	TACAA	0.04	1.18×10^−1^	0.64 [0.36–1.12]
	TGCAA	0.86	2.62×10^−1^	1.17 [0.89–1.54]

Note: SNPs in CD case-control sample and omnibus p-values for association with CD susceptibility. P-values<0.05 are depicted in bold (uncorrected p-values). No association remained significant after Bonferroni correction for multiple testing. rs10065172, which is in linkage disequilibrium with rs13361189, was excluded from the haplotype analysis.

### Genotype-phenotype analysis

We further investigated whether *IRGM* SNPs are associated with certain phenotypic characteristics in IBD patients. Based on the Montreal classification of IBD, the phenotypic data of IBD patients were analyzed for anatomic localization. However, none of the *IRGM* SNPs investigated were associated with specific disease localization in CD (Table S6) or UC (Table S7). Moreover, a detailed genotype-phenotype analysis in CD patients of the exonic synonymous SNP rs10065172 = p.Leu105Leu, which was in linkage disequilibrium with rs13361189 and with the previously identified 20-kb deletion polymorphism immediately upstream of *IRGM* (r^2^ = 1.0), did not reveal any significant associations with the CD phenotype (Table S8).

### Analysis for epistasis of IRGM with other major CD susceptibility genes

Finally, we analyzed potential evidence for gene-gene interactions of *IRGM* variants with other CD susceptibility genes such as variants in the *NOD2*, *IL23R* and *ATG16L1* gene including their effect on CD susceptibility. Interestingly, there was evidence for weak gene-gene-interaction between several SNPs of the two autophagy genes *IRGM* and *ATG16L1* (*ATG16L1* rs12471449, *ATG16L1* rs1441090, *ATG16L1* rs4663396), which, however, did not remain significant after Bonferroni correction (Table 5). The odds ratios of gene-gene interactions, which were significant before Bonferroni correction, are given in Table 6. There was no epistasis between *IRGM* and the other two major CD susceptibility genes *NOD2* and *IL23R*.

**Table 5:** Analysis for gene-gene interaction (epistasis) of *IRGM* SNPs with *NOD2*, *ATGT16L1*, and *IL23R* gene variants regarding CD susceptibility.

	rs13361189	rs4958847	rs1000113	rs11747270	rs931058
*NOD2* rs2066844 = p.Arg702Trp	8.10×10^−1^	5.00×10^−1^	8.75×10^−1^	8.24×10^−1^	7.87×10^−1^
*NOD2* rs2066845 = p.Gly908Arg	2.07×10^−1^	2.37×10^−1^	1.55×10^−1^	5.97×10^−1^	4.33×10^−1^
*NOD2* rs2066847 = p.Leu1007fsX1008	9.43×10^−1^	9.70×10^−1^	9.72×10^−1^	6.26×10^−1^	8.65×10^−1^
*ATG16L1* rs13412102	4.14×10^−1^	3.40×10^−1^	6.24×10^−1^	7.41×10^−1^	4.57×10^−1^
*ATG16L1* rs12471449	**2.00×10^−2^**	6.09×10^−2^	**2.01×10^−2^**	1.11×10^−1^	**5.41×10^−3^**
*ATG16L1* rs6431660	8.53×10^−1^	8.42×10^−1^	8.38×10^−1^	4.77×10^−1^	7.64×10^−1^
*ATG16L1* rs1441090	1.27×10^−1^	6.74×10^−1^	**4.45×10^−2^**	1.35×10^−1^	**2.85×10^−0^**
*ATG16L1* rs2289472	9.53×10^−1^	6.58×10^−1^	8.63×10^−1^	5.34×10^−1^	6.79×10^−1^
*ATG16L1* rs2241880	6.15×10^−1^	5.88×10^−1^	6.01×10^−1^	4.72×10^−1^	9.62×10^−1^
*ATG16L1* rs2241879	9.24×10^−1^	7.94×10^−1^	9.71×10^−1^	6.19×10^−1^	7.13×10^−1^
*ATG16L1* rs3792106	7.19×10^−1^	9.08×10^−1^	5.54×10^−1^	4.53×10^−1^	9.29×10^−1^
*ATG16L1* rs4663396	**1.19×10^−2^**	**2.89×10^−3^**	**1.91×10^−2^**	1.94×10^−1^	**2.16×10^−2^**
*IL23R* rs1004819	6.75×10^−1^	2.61×10^−1^	5.01×10^−1^	7.05×10^−1^	6.52×10^−1^
*IL23R* rs7517847	1.36×10^−1^	3.36×10^−1^	1.33×10^−1^	4.30×10^−1^	8.05×10^−2^
*IL23R* rs10489629	6.60×10^−1^	9.74×10^−1^	9.49×10^−1^	8.73×10^−2^	2.74×10^−1^
*IL23R* rs2201841	2.66×10^−1^	5.59×10^−2^	8.60×10^−2^	5.72×10^−2^	2.54×10^−1^
*IL23R* rs11465804	6.04×10^−2^	1.09×10^−1^	1.98×10^−1^	9.85×10^−1^	1.76×10^−1^
*IL23R* rs11209026 = p.Arg381Gln	1.93×10^−1^	1.62×10^−1^	3.92×10^−1^	6.84×10^−1^	2.60×10^−1^
*IL23R* rs1343151	7.70×10^−1^	3.64×10^−1^	9.57×10^−1^	3.71×10^−1^	9.69×10^−1^
*IL23R* rs10889677	1.63×10^−1^	**3.56×10^−2^**	6.47×10^−2^	**3.27×10^−2^**	1.79×10^−1^
*IL23R* rs11209032	2.66×10^−1^	1.80×10^−1^	1.92×10^−1^	8.23×10^−2^	2.84×10^−1^
*IL23R* rs1495965	8.54×10^−1^	4.66×10^−1^	6.00×10^−1^	1.79×10^−1^	9.86×10^−1^

Note: p-values for epistasis between *NOD2* and *ATGT16L1* SNPs and *IRGM* SNPs in CD case-control sample. P-values<0.05 are depicted in bold. After Bonferroni correction, none of the associations highlighted in bold remained significant. We excluded rs10065172, which is in linkage disequilibrium with rs13361189, from the epistasis analysis.

**Table 6:** Odds ratios (ORs) and 95% CI for the gene-gene interaction (epistasis) found to be significant (before Bonferroni correction) for CD susceptibility (shown in Table 5).

	rs13361189	rs10065172	rs4958847	rs1000113	rs11747270	rs931058
***ATG16L1*** ** rs12471449**	1.87 [1.10–3.17]	1.92 [1.12–3.30]	n.s.	1.91 [1.11–3.29]	n.s.	2.21 [1.26–3.87]
***ATG16L1*** ** rs1441090**	n.s.	n.s.	n.s.	2.35 [1.02–5.39]	n.s.	2.42 [2.00–5.33]
***ATG16L1*** ** rs4663396**	1.80 [1.14–2.83]	1.76 [1.11–2.78]	1.81 [1.22–2.66]	1.76 [1.10–2.82]	n.s.	1.75 [1.09–2.81]
***IL23R*** ** rs10889677**	n.s.	n.s.	0.72 [0.54–0.98]	n.s.	0.58 [0.35–0.96]	n.s.

Note: After Bonferroni correction, significance was lost for all gene-gene interactions shown in this table. rs10065172, which is in linkage disequilibrium with rs13361189, was excluded from the epistasis analysis.

n.s.: non-significant interactions (not shown).

## Discussion

This study represents a detailed analysis of *IRGM* gene variants regarding their role in the susceptibility and phenotype of IBD in a large cohort of more than 2000 Caucasian individuals. In line with previous GWAS and replication studies [12], [13], [14], [15], [16], [17], [18], [19], our results confirm an association of the *IRGM* variant rs13371189 with CD susceptibility. A detailed functional study identified a deletion polymorphism directly upstream of the *IRGM* locus as a candidate SNP to explain the CD association at this locus [22], affecting the tissue-specific expression level of IRGM [37]. This 20-kb deletion polymorphism is in perfect linkage disequilibrium (r^2^ = 1.0) with SNP rs13361189, therefore implicating that rs13361189 is a proxy for this deletion polymorphism.

Similar to the study by McCarroll et al. [22], we demonstrate that the common exonic synonymous SNP rs10065172 = p.Leu105Leu is in linkage disequilibrium with rs13361189 and therefore also with the previously identified 20-kb deletion polymorphism. The exonic SNP rs10065172 (c.313C>T) has been previously classified as non-causative given the absence of an alteration in the IRGM protein sequence or splice sites, although this view is challenged by the results of a very recent study [21]. The study by Brest et al. demonstrated that a family of microRNAs (miRNAs), miR-196, is overexpressed in the inflamed intestinal epithelium of CD patients and downregulates the *IRGM* protective variant (c.313C) but not the CD-associated allele (c.313T) [21]. The same study demonstrated that the resulting loss of tight regulation of IRGM expression compromises the control of the intracellular replication of CD-associated adherent invasive *Escherichia coli* (AIEC) by affecting the efficacy of bacterial phagocytosis (xenophagy) [21]. Therefore, Brest et al. [21] suggest the synonymous SNP rs10065172 (c.313C>T) as a likely causal variant. rs10065172 has been also shown to be associated with susceptibility to tuberculosis [38] which is of interest, given evidence that certain mycobacteria may play a role in the pathogenesis of CD. Moreover, a functional study demonstrated that IRGM induces autophagy to eliminate intracellular mycobacteria [23].

Overall, the association signal of *IRGM* with CD found in our study was considerably weaker than that shown by us for the other autophagy gene *ATG16L1* in a similar sized cohort [11]. Similarly, the recent CD meta-analyses showed a stronger association signal for *ATG16L1* than for *IRGM* [5]. Anderson et al. performed a very large meta-analysis of CD and UC associated susceptibility loci [6]. In this analysis, the CD case-control cohort included n = 6,333 CD patients and n = 15,056 controls, while the UC case-control cohort consisted of n = 6,687 UC patients and 19,718 controls [6]. In this large meta-analysis, they demonstrated that the *IRGM* SNP rs7714584 is associated with both CD (p = 7.76×10^−19^; OR 1.37, 95% CI 1.28–1.47) and UC (p = 3.95×10^−4^; OR 1.14, 95% CI 1.06–1.22) [6]. However, the authors defined only SNPs with p-values of <1×10^−4^ to be significantly associated with both CD and UC and therefore included *IRGM* not in the list of susceptibility loci shared between CD and UC [6]. In addition, the meta-analysis of Palomino-Morales et al. demonstrated also an association of two *IRGM* SNPs with UC (rs13361189 p = 0.0069, pooled OR = 1.16; rs4958847 p = 0.014, pooled OR = 1.13) [14]. Most importantly, a recent very large IBD meta-analysis comprising 75,000 IBD patients and controls confirmed the *IRGM* gene region to be strongly associated with CD (p = 2.94×10^−37^) and to a much lesser degree with UC (p = 0.0025 for the UC GWAS cohort and p = 1.05×10^−7^ for the combined UC Immunochip and GWAS cohort) [39]. Therefore, based on these meta-analyses as well as on our results showing a trend for association with UC for the *IRGM* SNP rs11747270 (p = 0.084, OR 1.42 [0.96–2.12] for comparing minor allele frequencies; p = 0.033, OR 5.34 [1.03–34.64] for comparing genotype frequencies; Table 2) and associations of several *IRGM* haplotypes with UC (p<0.05), *IRGM* can be regarded to be weakly associated with UC and as a shared susceptibility gene of both UC and CD, although it has a much more prominent role in the pathogenesis of CD.

In addition, we also performed a detailed genotype-phenotype analysis of *IRGM* variants in CD and UC patients. In contrast to a recent study of Latiano et al. [40] demonstrating an association of *IRGM* variants with fistulizing CD, our genotype-phenotype analysis did not reveal any significant association of *IRGM* variants with the CD phenotype. We were also unable to confirm an association with ileal CD found in a previous study of a smaller CD cohort from New Zealand [18]. Our findings may be related to the rather weak association signal found for *IRGM* in the German CD cohort, although the results of this genotype-phenotype analysis are consistent with the lack of a well-defined phenotype in CD patients carrying risk alleles of the other autophagy gene *ATG16L1* [11].

The identification of the two major CD susceptibility genes *ATG16L1* and *IRGM* involved in autophagy has significantly strengthened the importance of autophagy and bacterial xenophagy in the complex and multifactorial etiology of IBD. However, potential epistatic interactions between *ATG16L1* and *IRGM* have not been investigated in detail so far. We therefore analyzed epistasis between these two genes, demonstrating a weak gene-gene-interaction between several SNPs of the two autophagy genes *IRGM* and *ATG16L1* which, however, did not remain significant after Bonferroni correction. Given their close functional relationship, this potential epistasis signal is highly interesting. Very recently, the largest IBD meta-analysis published so far (including 75,000 IBD patients and controls) was made publicly available [39]. Part of this meta-analysis was an epistasis analysis in IBD, UC and CD datasets of the Immunochip study. While the analyses of the CD and UC subsets were inconclusive, the results for the analysis with IBD showed only one suggestive result between SNPs near *SLC7A10* (rs17694108) and *IL2RA* (rs12722515) with a p-value of 3.26×10^−5^ [39]. Therefore, the weak gene-gene interaction found between *IRGM* and *ATG16L1* regarding CD susceptibility in our study, which was not significant after Bonferroni correction, could not been replicated on a significant level in this very large CD cohort. Thus, it is unlikely that epistasis between the two major autophagy genes contributes significantly to CD susceptibility.

There is increasing evidence for important intersections of autophagy and intracellular bacterial sensing (demonstrated by the importance of *NOD2* in autophagy induction [41], [42]) in the pathogenesis of IBD. Moreover, recent studies identified a new pathway closely linked to autophagy and innate immunity, which is characterized by an unfolded protein response, stimulated by endoplasmic reticulum (ER) stress due to the accumulation of misfolded proteins. Several genes involved in ER stress, including *XBP1* and *ORMDL3* have been linked to the IBD pathogenesis on a genetic level [43], [44]. Interestingly, *ATG16L1*, *NOD2*, and *XBP1* have been also demonstrated to affect the function of Paneth cells [43], [45], [46], suggesting a central role for this cell type in the development of IBD.

These recent findings are in line with raising evidence that NOD2 is involved in regulation of autophagy. Dendritic cells from CD patients expressing CD-associated *NOD2* or *ATG16L1* variants were shown to be defective in autophagy induction, bacterial trafficking and antigen presentation [41]. Most interestingly, a recent study demonstrated that the intracellular sensors NOD1 and NOD2 are critical for the autophagic response to invasive bacteria by recruiting the autophagy protein ATG16L1 to the plasma membrane at the bacterial entry site [42]. In cells homozygous for the CD-associated *NOD2* frameshift mutation (p.Leu1007fsX1008), mutant NOD2 failed to recruit ATG16L1 to the plasma membrane and wrapping of invading bacteria by autophagosomes was impaired [42]. This is of particular interest, since we previously demonstrated a very severe stricturing phenotype in CD patients homozygous for the *NOD2* p.Leu1007fsX1008 mutation associated with early disease onset, ileal stenosis, recurrent need for surgery and increased prevalence of entero-enteral fistulae [32], [33]. However, despite the central functional role of *NOD2* in the induction of autophagic processes, our study could not demonstrate gene-gene-interactions between *NOD2* and *IRGM* regarding CD susceptibility. Moreover, we could not identify significant epistatic interactions between *IRGM* and *IL23R*, the main IBD susceptibility gene involved in Th17 responses. Of interest, a very recent study demonstrated *IL23R* variants as susceptibility variants for leprosy and suggested a potential involvement of *IL23R* in the autophagocytosis of mycobacteria involved in the pathogenesis of leprosy [47].

In conclusion, our results confirm *IRGM* as susceptibility gene for CD in the German population, while we did not show an association with a specific IBD subphenotype. The strongest association signals for CD susceptibility were found for rs13361189 (proxy for the common, 20-kb deletion polymorphism upstream of *IRGM*) and the exonic synonymous SNP rs10065172 = p.Leu105Leu, supporting previous functional studies that these two SNPs may be the causal variants. However, the strength of the association signal with CD found here was several log-fold weaker than that demonstrated by us for the second autophagy gene *ATG16L1* [11], suggesting a more important role for *ATG16L1* in the CD pathogenesis. In UC, several *IRGM* haplotypes were weakly associated with UC susceptibility. This is consistent with recent meta-analyses which found weak associations with UC but very strong disease associations with CD. One might therefore hypothesize that autophagy genes such as *IRGM* and *ATG16L1* play a more important role in the susceptibility to CD than UC. The potential epistasis signal between *IRGM* and *ATG16L1* regarding CD susceptibility found in this study is highly interesting but could not been confirmed in a very large recent IBD meta-analysis [39] arguing against a major role of epistasis between *IRGM* and *ATG16L1* regarding CD susceptibility.

## Supporting Information

Table S1 **Primer sequences and FRET probe sequences used for genotyping** ***IRGM*** **variants.**
(DOC)

Table S2 **Primer sequences used for the sequence analysis of** ***IRGM*** **variants.**
(DOC)

Table S3 **Analysis for linkage disequilibrium in CD patients.** Values are given as r^2^/D′-measurements.
(DOC)

Table S4 **Analysis for linkage disequilibrium in UC patients.** Values are given as r^2^/D′-measurements.
(DOC)

Table S5 **Analysis for linkage disequilibrium in controls.** Values are given as r^2^/D′-measurements.
(DOC)

Table S6 **P-values for allelic association of** ***IRGM*** **gene markers with the anatomic location of Crohn's disease (CD) according to the Montreal classification.**
(DOC)

Table S7 **P-values for allelic associations of** ***IRGM*** **gene markers with the anatomic location of ulcerative colitis (UC) according to the Montreal classification.**
(DOC)

Table S8 **Genotype-phenotype-analysis of the exonic synonymous SNP rs10065172 = p.Leu105Leu.**
(DOC)
