# Supplementary material for: IRGM Variants and Susceptibility to Inflammatory Bowel Disease in the German Population
Source: PLoS One. 2013 Jan 24;8(1):e54338. doi: 10.1371/journal.pone.0054338 (PMC3554777; doi:10.1371/journal.pone.0054338)
Supplement: Table S1 — Primer sequences and FRET probe sequences used for genotyping IRGM variants. (DOC) [file pone.0054338.s001.doc]

**Table S1.** Primer sequences and FRET probe sequences used for genotyping *IRGM* variants.

| **Polymorphism** | **Primer sequences** | **FRET probe sequences** |
| --- | --- | --- |
| rs13361189 | F: GCTGAGCACGGGGTCTAC | LC640-CTTGAAAAT^+^CGGAT^+^GTAT^+^ATTAG |
|  | R: GCTGACCTCCCACACTCAC | GGCCCGTGTCGTACCCAAGCAGAGTGT-FL |
| rs10065172 | F: TTGGCAAGCATCACATGATT | GGAGAACTACCTGATGGAAATGC-FL |
|  | R: TCACCTCCTACTGAGCTGGTAA | LC670-GTTCAACCGGTATGACTTCATCATGGTTGC |
| rs4958847 | F: AAGGTACAAAAATGTTATAGGAGACG | CACCTCTCACTGGGAGAAGCTTTATAGATTTC-FL |
|  | R: TCGGTGGTGATATCCCCTT | LC670-TTGCCCAATATAGCTAAATAATGC |
| rs1000113 | F: GTGTGATGCAGAGGCACTAAG | TACAAAATAACAGCATACACATGG-FL |
|  | R: GGTGTTTTCTTGGCACTGATG | LC610-CATTGCAATCAAGTATATTGGAGTTATTAGGAT |
| rs11747270 | F: GCTGAGATGGATGTCAGTGTTAG | TAGATGAGGTCTGTGTTACATAAATG-FL |
|  | R: TGGCCAGACTAAGTACTTGGC | LC670-GCATTGAAACACCAGTGTCAATCAGAAAAAT |
| rs931058 | F: CTAATAAGTTACTGGTGGCATGA | GTACAAAAATGTATGCATTCAGAAAT-FL |
|  | R: GGGCTCAAGCAATCCTCTAGC | LC670-TAGTCCAGGCATGGTGGCTTATGCCT |

Note: F: forward primer, R: reverse Primer, FL: Fluorescein, LC610: LightCycler-Red 610; LC640: LightCycler-Red 640. The polymorphic position within the sensor probe is underlined. A phosphate is linked to the 3'-end of the acceptor probe to prevent elongation by the DNA polymerase in the PCR. T^+^ indicates LNA modification to incresse melting temperature.
