# Supplementary material for: IRGM Variants and Susceptibility to Inflammatory Bowel Disease in the German Population
Source: PLoS One. 2013 Jan 24;8(1):e54338. doi: 10.1371/journal.pone.0054338 (PMC3554777; doi:10.1371/journal.pone.0054338)
Supplement: Table S2 — Primer sequences used for the sequence analysis of IRGM variants. (DOC) [file pone.0054338.s002.doc]

**Table S2.** Primer sequences used for the sequence analysis of *IRGM* variants.

| **Polymorphism** | **Primer sequences** |
| --- | --- |
| rs13361189 | CATTAAACTCTTTACCATTGTACTCCT |
|  | AGCAGACAGGTTTGAAGATGC |
| rs10065172 | CAATGTAGAACTTCTTTCCCATGTC |
|  | TCACCTCCTACTGAGCTGGTAA |
| rs4958847 | AAGGTACAAAAATGTTATAGGAGACG |
|  | TCGGTGGTGATATCCCCTT |
| rs1000113 | GTGTGATGCAGAGGCACTAAG |
|  | GGTGTTTTCTTGGCACTGATG |
| rs11747270 | GCTGAGATGGATGTCAGTGTTAG |
|  | AATTACCAATAAATGCAAAGAGAATC |
| rs931058 | GTGGTTTATGTTAACTGCTGTGA |
|  | GGGCTCAAGCAATCCTCTAGC |
