# Supplementary material for: IRGM Variants and Susceptibility to Inflammatory Bowel Disease in the German Population
Source: PLoS One. 2013 Jan 24;8(1):e54338. doi: 10.1371/journal.pone.0054338 (PMC3554777; doi:10.1371/journal.pone.0054338)
Supplement: Table S3 — Analysis for linkage disequilibrium in CD patients. Values are given as r2/D′-measurements. (DOC) [file pone.0054338.s003.doc]

**Table S3.** Analysis for linkage disequilibrium in CD patients. Values are given as r²/D'-measurements.

|  | **rs13361189** | **rs10065172** | **rs4958847** | **rs1000113** | **rs11747270** | **rs931058** |
| --- | --- | --- | --- | --- | --- | --- |
| rs13361189 | * | * | * | * | * | * |
| rs10065172 | 0.99/0.99 | * | * | * | * | * |
| rs4958847 | 0.71/0.98 | 0.71/0.98 | * | * | * | * |
| rs1000113 | 0.90/0.99 | 0.95/0.99 | 0.67/1.00 | * | * | * |
| rs11747270 | 0.53/0.99 | 0.54/1.00 | 0.38/0.98 | 0.53/0.94 | * | * |
| rs931058 | 0.59/0.84 | 0.60/0.85 | 0.43/0.84 | 0.67/0.85 | 0.38/0.77 | * |
