# Supplementary material for: IRGM Variants and Susceptibility to Inflammatory Bowel Disease in the German Population
Source: PLoS One. 2013 Jan 24;8(1):e54338. doi: 10.1371/journal.pone.0054338 (PMC3554777; doi:10.1371/journal.pone.0054338)
Supplement: Table S5 — Analysis for linkage disequilibrium in controls. Values are given as r2/D′-measurements. (DOC) [file pone.0054338.s005.doc]

**Table S5. Analysis for linkage disequilibrium in controls. Values are given as r²/D'-measurements.**

|  | **rs13361189** | **rs10065172** | **rs4958847** | **rs1000113** | **rs11747270** | **rs931058** |
| --- | --- | --- | --- | --- | --- | --- |
| rs13361189 | * | * | * | * | * | * |
| rs10065172 | 0.98/0.99 | * | * | * | * | * |
| rs4958847 | 0.64/0.99 | 0.62/0.98 | * | * | * | * |
| rs1000113 | 0.91/0.98 | 0.91/0.97 | 0.61/1.00 | * | * | * |
| rs11747270 | 0.57/0.96 | 0.58/0.96 | 0.36/0.95 | 0.52/0.89 | * | * |
| rs931058 | 0.61/0.79 | 0.61/0.78 | 0.40/0.79 | 0.65/0.82 | 0.33/0.73 | * |
