# Supplementary material for: IRGM Variants and Susceptibility to Inflammatory Bowel Disease in the German Population
Source: PLoS One. 2013 Jan 24;8(1):e54338. doi: 10.1371/journal.pone.0054338 (PMC3554777; doi:10.1371/journal.pone.0054338)
Supplement: Table S6 — P-values for allelic association of IRGM gene markers with the anatomic location of Crohn's disease (CD) according to the Montreal classification. (DOC) [file pone.0054338.s006.doc]

**Table S6.** P-values for allelic association of *IRGM* gene markers with the anatomic location of Crohn’s disease (CD) according to the Montreal classification [26].

| **Anatomic location** | **rs13361189** | **rs10065172** | **rs4958847** | **rs1000113** | **rs11747270** | **rs931058** |
| --- | --- | --- | --- | --- | --- | --- |
| **L1** (ileal) | 7.87 x 10 ^-1^ | 2.64 x 10 ^-1^ | 6.93 x 10 ^-1^ | 4.53 x 10 ^-1^ | 9.90 x 10 ^-1^ | 4.95 x 10 ^-1^ |
| *(n=113)* |  |  |  |  |  |  |
| **L2** (colonic) | 9.17 x 10 ^-1^ | 3.56 x 10 ^-1^ | 3.83 x 10 ^-1^ | 2.97 x 10 ^-1^ | 3.90 x 10 ^-1^ | 4.94 x 10 ^-1^ |
| *(n=98)* |  |  |  |  |  |  |
| **L3** (ileocolonic) | 9.90 x 10 ^-1^ | 9.99 x 10 ^-1^ | 8.88 x 10 ^-1^ | 7.78 x 10 ^-1^ | 6.71 x 10 ^-1^ | 9.31 x 10^-1^ |
| *(n=550)* |  |  |  |  |  |  |
| **L1+L3** (Any ileal involvement) | 8.22 x 10 ^-1^ | 8.16 x 10 ^-1^ | 6.03 x 10 ^-1^ | 7.44 x 10 ^-1^ | 6.17 x 10 ^-1^ | 6.16 x 10^-1^ |
| *(n=648)* |  |  |  |  |  |  |
