# Supplementary material for: IRGM Variants and Susceptibility to Inflammatory Bowel Disease in the German Population
Source: PLoS One. 2013 Jan 24;8(1):e54338. doi: 10.1371/journal.pone.0054338 (PMC3554777; doi:10.1371/journal.pone.0054338)
Supplement: Table S7 — P-values for allelic associations of IRGM gene markers with the anatomic location of ulcerative colitis (UC) according to the Montreal classification. (DOC) [file pone.0054338.s007.doc]

**Table S7.** P-values for allelic associations of *IRGM* gene markers with the anatomic location of ulcerative colitis (UC) according to the Montreal classification [26].

| **Anatomic location** | **rs13361189** | **rs10065172** | **rs4958847** | **rs1000113** | **rs11747270** | **rs931058** |
| --- | --- | --- | --- | --- | --- | --- |
| **E1** (ulcerative proctitis) | 3.79 x 10 ^-1^ | 4.34 x 10 ^-1^ | 1.35 x 10 ^-1^ | 2.98 x 10 ^-1^ | 6.66 x 10 ^-1^ | 8.74 x 10 ^-2^ |
| *(n=24)* |  |  |  |  |  |  |
| **E2** (left-sided UC) | 2.14 x 10 ^-1^ | 2.07 x 10 ^-1^ | 2.28 x 10 ^-1^ | 2.16 x 10 ^-1^ | 7.29 x 10 ^-2^ | 1.76 x 10 ^-1^ |
| *(n=96)* |  |  |  |  |  |  |
| **E3** (pancolitis) | 1.87 x 10 ^-1^ | 1.95 x 10 ^-1^ | 1.01 x 10 ^-1^ | 2.19 x 10 ^-1^ | 1.12 x 10 ^-1^ | 1.27 x 10 ^-1^ |
| *(n=140)* |  |  |  |  |  |  |
