# Supplementary material for: IRGM Variants and Susceptibility to Inflammatory Bowel Disease in the German Population
Source: PLoS One. 2013 Jan 24;8(1):e54338. doi: 10.1371/journal.pone.0054338 (PMC3554777; doi:10.1371/journal.pone.0054338)
Supplement: Table S8 — Genotype-phenotype-analysis of the exonic synonymous SNP rs10065172 = p.Leu105Leu. (DOC) [file pone.0054338.s008.doc]

**Table S8.** Genotype-phenotype-analysis of the exonic synonymous *IRGM* SNP rs10065172=p.Leu105Leu.

| **rs10065172=p.Leu105Leu** | **(1) CC** n=650 | **(2) CT** *n=156* | **(3) TT** *n=8* | **PT** | **ORT [95% CI]** |
| --- | --- | --- | --- | --- | --- |
| **Age at diagnosis** (yr) |  |  |  | 0.067 | 1.42 [0.98-2.07] |
| Mean ± SD | 28.15 ± 11.87 | 26.98 ± 12.13 | 31.75 ± 19.07 |  |  |
| Range | 6-78 | 7-71 | 17-73 |  |  |
| **Age at diagnosis** *(n=727)* |  |  |  |  |  |
| <=16 years (A1) *(n=81)* | 65 | 16 | 0 | 0.998 | 1.00 [0.56-1.80] (A1 vs. A2) |
| 17-40 years (A2) *(n=547)* | 439 | 102 | 6 | 0.406 | 1.28 [0.72-2.27] (A2 vs. A3) |
| > 40 years (A3) *(n=99)* | 83 | 14 | 2 | 0.531 | 1.28 [0.59-2.74] |
| **Location** (*n=770)* |  |  |  |  |  |
| Terminal ileum (L1) *(n=113)* | 89 | 23 | 1 | 0.739 | 1.09 [0.67-1.77] |
| Colon (L2) *(n=98)* | 82 | 15 | 1 | 0.322 | 0.75 [0.43-1.32] |
| Ileocolon (L3) *(n=549)* | 439 | 104 | 6 | 0.964 | 0.99 [0.67-1.46] |
| Upper GI (L4) *(n=10)* | 7 | 3 | 0 | 0.436 | 1.72 [0.44-6.73] |
| **Behaviour** ^1^ *(n=692)* |  |  |  |  |  |
| Non-stricturing - non-penetrating (B1) *(n=173)* | 138 | 34 | 1 | 0.955 | 1.01 [0.66-1.55] |
| Stricturing (B2) *(n=188)* | 144 | 42 | 2 | 0.189 | 1.31 [0.88-1.94] |
| Penetrating (B3) *(n=331)* | 268 | 59 | 4 | 0.524 | 0.89 [0.62-1.28] |
| **Use of immunosuppressive agents** ^2^ *(n=585)* | No: 92 | 14 | 0 | 0.126 | 1.60 [0.88-2.94] |
|  | Yes: 385 | 88 | 6 |  |  |
| **Surgery because of CD** ^3^*(n=723)* | No: 253 | 60 | 2 | 0.955 | 1.01 [0.70-1.46] |
|  | Yes: 327 | 75 | 6 |  |  |
| **Fistulas** *(n=737)* | No: 274 | 73 | 3 | 0.293 | 0.82 [0.57-1.18] |
|  | Yes: 315 | 67 | 5 |  |  |
| **Stenosis** *(n=739)* | No: 214 | 45 | 2 | 0.311 | 1.22 [0.83-1.79] |
|  | Yes: 377 | 95 | 6 |  |  |

Note: rs10065172 is in perfect linkage disequilibrium with rs13361189 and with the previously identified 20-kb deletion polymorphism, immediately upstream of *IRGM* (r^2^=1.0) and therefore a proxy for these two other *IRGM* SNPs. *P*-value for testing for differences between carriers and non-carriers of the T allele. ORT: corresponding odds ratios and 95% confidence intervals (95% CI). For age at diagnosis, *P*-values are given based on a median split.

1 Disease behaviour was defined according to the Montreal classification. A stricturing disease phenotype was defined as presence of stenosis without penetrating disease. The diagnosis of stenosis was made surgically, endoscopically, or radiologically (using MRI enteroclysis).

2 Immunosuppressive agents included azathioprine, 6-mercaptopurine, methotrexate, infliximab and/or adalimumab.

3 Only surgery related to CD-specific problems (e.g. fistulectomy, colectomy, ileostomy) was included.
